# The role of discrimination in the relation between COVID-19 sequelae, psychological distress, and work impairment in COVID-19 survivors

**DOI:** 10.1038/s41598-022-26332-6

**Published:** 2022-12-23

**Authors:** Shinya Ishii, Aya Sugiyama, Noriaki Ito, Kei Miwata, Yoshihiro Kitahara, Mafumi Okimoto, Akemi Kurisu, Kanon Abe, Hirohito Imada, Tomoyuki Akita, Tatsuhiko Kubo, Akira Nagasawa, Toshio Nakanishi, Toshiro Takafuta, Masao Kuwabara, Junko Tanaka

**Affiliations:** 1 Department of Medicine for Integrated Approach to Social Inclusion, Graduate School of Biomedical and Health Sciences, Hiroshima University, 1-2-3 Kasumi, Minami-ku, Hiroshima, 734-8551 Japan; 2 Department of Epidemiology, Infectious Disease Control and Prevention, Graduate School of Biomedical and Health Sciences, Hiroshima University, 1-2-3, Kasumi, Minami-ku, Hiroshima, 734-8551 Japan; 3 Hiroshima City Funairi Citizens Hospital, Hiroshima, Japan; 4 Department of Public Health and Health Policy, Graduate School of Biomedical and Health Sciences, Hiroshima University, Hiroshima, Japan; 5 Miyoshi Central Hospital, Hiroshima, Japan; 6 Hiroshima Prefectural Center for Disease Control and Prevention, Hiroshima, Japan

**Keywords:** Epidemiology, Psychology

## Abstract

Perceived discrimination and work impairment are commonly observed in COVID-19 survivors, but their relationship has not been well understood. We aimed to evaluate the role of discrimination in the development of psychological distress and work impairment in COVID-19 survivors. From April 2020 to November 2021, 309 patients were recruited at two designated COVID-19 hospitals in Japan. Participants completed a standardized questionnaire including COVID-19 sequelae, psychological distress, impairments in work performance and perceived discrimination. The majority of participants (62.5%) experienced one or more COVID-19 sequelae. Psychological distress was observed in 36.9% and work impairment in 37.9%. In multivariate logistic regression analyses, COVID-19 sequelae and discrimination were associated with both psychological distress and work impairment. Mediation analysis demonstrated that the direct effect of sequelae on work impairment was non-significant after accounting for psychological distress, suggesting that the effect of sequelae on work impairment was mainly mediated through psychological distress. These findings were replicated in a subgroup analysis limited to patients with mild COVID-19. We conclude that discrimination plays an important role in the development of psychological distress and work impairment, and that both discrimination and psychological distress should be targets of intervention in COVID-19 survivors.

## Introduction

Acute coronavirus disease 2019 (COVID-19) is an infectious disease caused by severe acute respiratory syndrome coronavirus 2 (SARS-CoV-2), and has led to a global pandemic. COVID-19 was initially considered to be a time-limited disease, albeit one with severe symptoms and dire outcomes such as high mortality. However, grave concerns have recently been raised about the long-term effects of COVID-19. Mounting evidence suggests that symptoms such as fatigue, shortness of breath, headaches, and anosmia/dysgeusia may persist for months, resulting in a substantial burden on COVID-19 survivors^1–3^. Some individuals may experience persistent neuropsychiatric symptoms such as cognitive dysfunction, depression, anxiety, reduced sleep quality, and post-traumatic stress^1,2,4–7^. In addition, a considerable proportion of COVID-19 patients, even those with mild cases, experience functional impairment in their work, social, and home life after recovery from the acute phase^8,9^. Some studies have shed light on the relationship between mental health and functional impairment in COVID-19 patients, demonstrating that COVID-19–related stress is associated with worsening anxiety, depression, and functional impairment^10,11^. Those with persistent symptoms after COVID-19 may have an increased utilization of healthcare resources and more functional impairment^12^.

COVID-19–related discrimination has been another major concern. As COVID-19 spread worldwide, fear of contagion and limited knowledge promoted negative attitudes or even discrimination towards people of Asian descent, due to the presumed origin of the virus, as well as towards those suspected to have or diagnosed with COVID-19^13,14^. Anti-Asian discrimination was widespread, particularly in Western countries^15–17^, and it negatively affected their mental and physical health and well-being^18–20^. Discrimination influenced their health behaviors as well, leading to low compliance with social-distancing recommendations^21,22^.

Discrimination also posed a serious threat to COVID-19 survivors^23,24^, and was shown to be associated with mental health problems such as psychological distress^25^, anxiety^26^, depression, difficulty sleeping^27^, and PTSD symptoms^14,28^. Hence, it is possible that discrimination against COVID-19 survivors adversely influences their mental health, which in turn impairs their social life. However, no studies have examined the relation between discrimination and functional impairment in this population.

It is imperative to understand the mechanisms underlying discrimination, mental health and associated functional impairment in COVID-19 in order to devise appropriate interventions to prevent or reduce the long-term adverse effects of the disease. This study aimed to elucidate the relationship between discrimination and COVID-19 sequelae, psychological distress, and work impairment in COVID-19 survivors in Hiroshima, Japan. We hypothesized that both COVID-19 sequelae and COVID-19–related discrimination would be risk factors for psychological distress and work impairment after COVID-19. We also hypothesized that psychological distress would mediate the relationship between COVID-19 sequelae and work impairment, i.e., the long-term effects of COVID-19 on work function would at least partially be explained by its mental effects. We additionally assessed whether discrimination moderated the indirect effect of sequelae on work impairment through psychological distress.

## Methods

### Participants and procedure

This cross-sectional study analyzed data collected in collaboration with two major COVID-19 hospitals (Hospital A and B) in Hiroshima Prefecture, Japan^29^. Hospital A is a designated medical institution for Class 2 infectious diseases and a regional hub medical center for mild and moderate COVID-19 in its catchment area. Hospital B is a designated key medical institution for COVID-19 patients in Hiroshima Prefecture. Patients who were diagnosed with COVID-19 based on polymerase chain reaction (PCR) testing and admitted to or visited the outpatient consultation clinics of these hospitals for COVID-19 were eligible. Patients who were admitted to Hospital A during April 14, 2020 and November 30, 2021 and visited its outpatient consultation clinic thereafter were recruited by their physicians. Patients who visited Hospital B between April 11, 2020, and May 1, 2020 were sent a letter requesting participation in the study in August 2020. Informed consent was obtained from all participants. A set of self-reported questionnaires was sent by mail to those who provided written consent to participate in the study after recovery from COVID-19.

### Measures

Standardized questionnaires were used to obtain the following information: age (years), gender, smoking history (current, ex-smoker, or never smoker), medical history, and COVID-19 sequelae (21 persistent symptoms, namely cough, fatigue, dyspnea, headache, palpitations, dry eye or mouth, olfactory dysfunction, dysgeusia, excess sputum, alopecia, nasal discharge, myalgia, sore throat, chest pain, abdominal pain, dizziness, arthralgia, anorexia, ocular hyperemia, insomnia, and loss of concentration). COVID-19-related experiences of discrimination were assessed by a multiple-choice question (possible responses: suffer from slander; being treated as infectious after recovery; work disadvantages caused by harmful rumors; trouble securing opportunities to train or study, including problems with commuting) supplemented by an open-ended question.

The Work Functioning Impairment Scale (WFun) was used to screen for impairments in work performance^30^. The Wfun questionnaire was developed to evaluate a worker’s health-related ability to complete their work tasks, and was demonstrated to be linearly associated with work productivity^31,32^. The scale comprises seven screening questions for measuring impaired work function, and responses are made on a 5-point scale from 1 to 5 (total score ranges from 7 to 35). A cutoff point of 14/15 was employed to indicate possible deficits in work performance^30^. Work impairment is graded by Wfun scores as follows: 14–20, mild; 21–27, moderate; and 28–35, severe.

The six-item Kessler Psychological Distress Scale (K6) was employed to assess the severity of psychological distress^33^. The Japanese version of the K6 Scale was demonstrated to have excellent discriminatory ability for mood and anxiety disorders^34^, and consists of six questions that are each scored from 0 to 4 (total score ranges from 0 to 24). Total scores of 0–4 are categorized as no distress, while those equal to or higher than 5 reflect possible mood or anxiety disorders^35^. The severity of these disorders corresponds to K6 scores as follows: 5–9, mild; 10–14, moderate; and 15–24, severe^35^.

The United States National Institutes of Health defines four levels of COVID-19 severity based on the need for respiratory support during hospitalization, as follows: no supplemental oxygen, require supplemental oxygen, require oxygen through non-invasive mechanical ventilation (non-IMV), and require IMV or ECMO (extracorporeal membrane oxygenation)^36^. For each patient, COVID-19 severity and the date of COVID-19 onset were obtained from medical records.

### Ethical approval

The authors assert that all procedures contributing to this work comply with the ethical standards of the relevant national and institutional committees on human experimentation and with the Helsinki Declaration of 1975, as revised in 2008. This study was conducted under the approval of the Ethical Committee for Epidemiology of Hiroshima University (Approval No. E-2122).

## Statistical analysis

Participant characteristics were summarized overall and according to the time of COVID-19 diagnosis and the presence or absence of COVID-19 sequelae. The distribution of SARS-CoV-2 variants in Hiroshima dramatically changed in March 2021, since the Alpha variant (B.1.1.7) became predominant after this time^37^. The characteristics of participants with and without COVID-19 sequelae were compared before and after March 1, 2021. The chi-square and Cochran–Armitage trend tests were used for categorical and ordered categorical variables, respectively, and the t-test was used for continuous variables (or the Wilcoxon rank-sum test if the distribution was skewed).

Multivariate logistic regression analysis was employed to identify factors associated with post–Covid-19 work impairment (defined as a Wfun score ≥ 14) and psychological distress (defined as K6 score ≥ 5). The independent variables were gender (male or female), age (< 40, 40–59, or ≥ 60 years), time period (before or after March 1, 2021), time from diagnosis to survey, and whether they received any support after diagnosis, presence or absence of the following: COVID-19 sequelae and COVID-19–related experience of discrimination.

Causal mediation analysis was performed to assess how COVID-19 sequelae influenced work impairment through psychological distress as a mediating factor^38^. The analysis was performed with bias-corrected confidence intervals using 5000 bootstrapping iterations. The sequential ignorability assumption was examined by a sensitivity analysis with various values for the correlation between mediator residuals and outcome regressions^39^. We also conducted a moderated mediation analysis to test the moderating effect of discrimination on the indirect effect of sequelae on work impairment through psychological distress by introducing the interaction terms with respect to sequelae and psychological distress to the logistic regression models.

Subgroup analyses limiting the analytic sample to participants with mild COVID-19 were conducted to examine the long-term effects of mild disease on work impairment and psychological distress.

All analyses were conducted using R version 4.1.12, and a p value less than 0.05 was considered statistically significant.

## Results

During the study period, 1177 patients were admitted to Hospital A and 329 (28.0%) agreed to participate in the study. Forty-three patients were sent a letter for participation and 22 (51.2%) responded to the survey. A total of 351 patients participated in this study and answered the survey. Those lacking information on COVID-19 severity (n = 10) or who did not fully complete the K6 (n = 7) or Wfun (n = 25) questionnaire were excluded from the analysis, resulting in an analytic sample of 309 participants.

### Participant characteristics

Table 1 summarizes the demographic and clinical characteristics of the study participants. The mean age was 47.0 years, with a standard deviation of 15.2 years. Approximately half of the participants (54.0%) were males. COVID-19 was classified as mild in the majority of participants (82.2%). Psychological distress (K6 score ≥ 5) was observed in 36.9% of the participants, while 37.9% exhibited work impairment (Wfun score ≥ 14). Questionnaire responses were collected at a median of 25 days [interquartile range 20–35]. At the time of the survey, 193 participants (62.5%) were experiencing one or more COVID-19 sequelae. The details of these sequelae are summarized in Supplementary Table 1.

**Table 1:** Characteristics of study participants by time period and presence or absence of COVID-19 sequelae.

	All	April 2020 to February 2021		*p*	March to November 2021		*p*
		With sequelae	Without sequelae		With sequelae	Without sequelae	
n	309	67	59		126	57	
Age	47.0 ± 15.2	51.0 ± 17.0	42.0 ± 16.3	0.003	45.8 ± 11.7	50.2 ± 16.8	0.079
**Gender**							
Male	54.0%	46.3%	55.9%	0.37	53.2%	63.2%	0.27
**COVID-19 severity**							
Mild	82.2%	77.6%	88.1%	0.07	84.1%	77.2%	0.38
Moderate	10.4%	14.9%	10.2%		7.1%	12.3%	
Severe	7.1%	6.0%	1.7%		8.7%	10.5%	
Critical	0.3%	1.5%	0		0	0	
Time from diagnosis to survey	25 [20, 35]	30 [21.5, 96]	29 [24.5, 131.5]	0.43	22 [20, 27]	24 [20, 30]	0.08
**Smoking**							
Never	51.8%	59.1%	60.3%	0.17	48.4%	42.1%	0.52
Former	31.1%	30.3%	19.0%		35.4%	35.1%	
Current	17.0%	10.6%	20.7%		16.1%	22.8%	
**K6 score**							
0–4	63.1%	55.2%	86.4%	< .001	55.6%	64.9%	0.12
5–9	22.0%	29.9%	10.2%		23.8%	21.1%	
10–14	8.4%	11.9%	1.7%		8.7%	10.5%	
15–24	6.5%	3.0%	1.7%		11.9%	3.5%	
**Wfun score**							
7–13	62.1%	56.7%	83.1%	< .001	53.2%	66.7%	0.091
14–20	14.9%	13.4%	10.2%		17.5%	15.8%	
21–27	10.0%	14.9%	5.1%		11.9%	5.3%	
≥ 28	12.9%	14.9%	1.7%		17.5%	12.3%	
Receiving no support	13.3%	9.0%	6.8%	0.75	18.3%	14.0%	0.62
Discrimination	32.7%	40.3%	49.2%	0.41	28.6%	15.8%	0.094

Mean and standard deviation are shown for continuous variables (or median and interquartile range if skewed) and percentages are shown for categorical variables.

*p* values were calculated using the t-test for continuous variables (or Wilcoxon rank-sum test if the distribution was skewed), and the chi-square and Cochran–Armitage trend tests for categorical and ordered categorical variables, respectively.

A total of 101 participants (32.7%) experienced COVID-19-related discrimination. The most common type of discrimination was “treated as infectious after recovery” (57.4%), followed by “work disadvantages caused by harmful rumors” (32.7%), “suffer from slander” (20.8%), and “trouble securing opportunities to train or study” (14.9%).

Between April 2020 and March 2021, participants with COVID-19 sequelae were significantly more likely to be older (*p* = 0.003) and to experience psychological distress and work impairment (both *p* < 0.001), but these differences were not statistically significant between March 2021 and November 2021.

### Psychological distress and work impairment

Tables 2 and 3 show the results of multivariate logistic regression analysis of psychological distress (K6 score ≥ 5) and work impairment (Wfun score ≥ 14) experienced after COVID-19. COVID-19 sequelae, infection after March 2021, and discrimination were independently and significantly associated with both psychological distress and work impairment, while female gender was associated with only psychological distress.

**Table 2:** Multivariate association between psychological distress and participant characteristics.

Characteristic	OR (95%CI)	*p*
**Age**		
Ref: < 40		
40–59	0.86 (0.48–1.53)	0.61
≥ 60	1.18 (0.57–2.44)	0.66
**Sex**		
Ref: male	2.11 (1.28–3.47)	0.004
Sequelae	2.36 (1.37–4.05)	0.002
**Time period**		
Ref: prior to 3/1/2021	1.94 (1.08–3.50)	0.026
Discrimination	2.10 (1.23–3.60)	0.007
Receiving no support	0.93 (0.44–1.93)	0.84
Time from diagnosis to survey	1.00 (0.99–1.01)	0.85

*OR* odds ratio, *CI* confidence interval.

**Table 3:** Multivariate association between work impairment and participant characteristics.

Variable	OR (95%CI)	p
**Age**		
Ref: < 40		
40–59	1.14 (0.64–2.04)	0.66
≥ 60	1.16 (0.55–2.45)	0.69
**Sex**		
Ref: male	1.30 (0.79–2.15)	0.31
Sequelae	2.44 (1.42–4.21)	0.001
**Time period**		
Ref: prior to 3/1/2021	2.40 (1.31–4.40)	0.005
Discrimination	3.42 (1.97–5.94)	< 0.001
Receiving no support	0.56 (0.26–1.21)	0.14
Time from diagnosis to survey	1.00 (0.997–1.001)	0.32

*OR* odds ratio, *CI* confidence interval.

Mediation analysis demonstrated that the effect of sequelae on the likelihood of work impairment was at least partially mediated through psychological distress. The effects of sequelae on psychological distress and of psychological distress on work impairment were both statistically significant (unstandardized coefficient 0.86 and 2.42, *p* = 0.002 and < 0.001, respectively). The bootstrapped indirect effect (unstandardized coefficient) was 0.09 with a 95% confidence interval between 0.02 and 0.15, suggesting that the indirect effect was statistically significant. The direct effect of sequelae on the likelihood of work impairment became non-significant in mediation analysis after accounting for psychological distress (unstandardized coefficient 0.09, *p* = 0.09). In moderated mediation analysis to test the moderating effect of discrimination on the indirect effect of sequelae on work impairment through psychological distress, the indirect effect did not significantly differ between participants with and without discrimination (*p* = 0.93).

#### Subgroup analysis

Among 254 participants who recovered from mild COVID-19 (82.2% of all participants), 158 (62.2%) reported COVID-19 sequelae; of these, 96 (37.8%) and 99 (39.0%) experienced psychological distress and work impairment, respectively. All analyses were repeated in analytic samples limited to participants with mild COVID-19, and the results replicated those obtained in the overall sample (Supplementary Tables 2 and 3, Supplementary Fig. 1).

## Discussion

In the present study, perceived discrimination was prevalent among COVID-19 survivors, and it was associated with both psychological distress and work impairment. COVID-19 sequelae were significantly and independently associated with both problems in analyses that treated each as separate outcomes, but mediation analysis demonstrated that the effect of sequelae on work impairment was mostly explained by their effect on psychological distress. The indirect effect of sequelae, i.e., the effect of sequelae on work impairment via psychological distress, was not moderated by discrimination.

The prevalence of COVID-19 sequelae varies greatly depending on the geographic location of the study area and on patient background factors^3^. A recent study in 63 hospitalized patients reported that the prevalence of sequelae was 76% at 14 days, 48% at 2 months, and 27% at 4 months after COVID-19 onset, results that were consistent with the prevalence of 62.5% in this study^40^.

Our finding that COVID-19 sequelae and discrimination were associated with psychological distress corroborated a previous study in which risk factors associated with anxiety and depression included perceived discrimination and a greater total number of symptoms in COVID-19 survivors^28^. We also showed that COVID-19 sequelae and discrimination were associated with functional limitations; to our knowledge, this finding has not been previously reported. We conducted additional analyses to delineate these associations and found that the effect of sequelae on work impairment was largely mediated via psychological distress. This suggests that interventions to prevent or reduce work impairment in COVID survivors should target psychological distress after recovery from COVID-19. For those who require hospitalization, discharge preparation programs can prepare them to effectively cope with persistent COVID-19 symptoms. Outpatient psychological support programs may provide close follow-up to monitor persistent symptoms of COVID-19 and psychological distress, and provide necessary care for them after discharge as well as for those with mild COVID-19 symptoms and recuperate without hospitalization. The development of such programs is urgently needed considering that the cumulative number of COVID-19 cases has been increasing worldwide, and a significant proportion of these individuals may suffer from COVID-19 sequelae, psychological distress, and functional impairment.

There is little information on the frequency of discrimination towards COVID-19 survivors in Japan. A small study in 27 patients showed that 23.1% of COVID-19 patients in Japan experienced discrimination or harassment after discharge^41^. Our study found a higher prevalence of perceived discrimination and suggests that discrimination towards COVID-19 survivors may be more widespread, possibly due to the continuation of the COVID-19 pandemic. Infection spread between individuals in close proximity may arguably intensify the fear of contagion, which could contribute to discrimination^42^. In the meantime, more knowledge and treatment options have rapidly become available, which can alleviate the fear of COVID-19. Change in virulence from COVID-19 variants over time may also affect peoples’ fear of and attitudes to COVID-19. Additional research is needed to follow longitudinal changes of perception towards COVID-19 patients. Our study demonstrated that although discrimination did not moderate the relationship between COVID-19 sequelae and work impairment, it remains an important risk factor for both psychological distress and work impairment. It is essential to implement measures to reduce discrimination against COVID-19 patients. Our findings in the overall sample were replicated in a subgroup analysis of participants who had recovered from mild COVID-19, implying that COVID-19 survivors may require support programs regardless of disease severity.

## Limitations

The study had several limitations. First, the study participants were recruited at two COVID-19 hospitals in Hiroshima, and our findings should be interpreted in the context of Japanese society and culture. When considering pandemics such as COVID-19, we must take into account cultural perceptions and ways they may affect symptom recognition, access to care, treatment provided, and fear of stigmatization^43^. Therefore, extrapolation of our findings to other societies with different cultural backgrounds should be carried out with caution. Second, the response rates at two hospitals were relatively low in this study though they are comparable to similar studies conducted during the COVID-19 outbreaks. It may introduce selection bias. Third, the results should be interpreted with much caution due to the relatively short duration between COVID-19 and data collection (median 25 days). Since many symptoms experienced 4 weeks after recovery from COVID-19 may become persistent for longer periods of time^44^ and functional impairment may be observed several months after COVID-19^8,9^, long-term effects of sequela on psychological distress and work impairment should be investigated in future research. The current study focuses on short-term impacts of COVID-19, which may provide insight into the development of psychological distress and work impairment after the acute phase of COVID-19 and help design interventions for early recognition and treatment. Fourth, the study design was cross-sectional and did not allow us to infer a causal relationship. The assumption of the mediation analysis was that the cause of work impairment was sequela and psychological distress was the mediator of their relationship. We have made this assumption based on previous studies reporting the association between persistent COVID-19 symptoms, stress and functional impairment^10–12^ and the presumed chronological order in which sequela develop first, psychological distress ensues, and returning to work after a certain quarantine period occurs at the end. However, it may be still possible that work impairment could cause increased psychological distress. The associations observed in this study should be replicated in future research using a longitudinal design. Fifth, discrimination was assessed using an unvalidated question since there was no specific scale to measure discrimination towards COVID-19 survivors. Finally, information on some important participant characteristics, such as a history of mental health disorders, was not available and could not be adjusted for.

## Conclusions

Discrimination affects approximately one-third of COVID-19 survivors and plays an important role in the development of psychological distress and work impairment. COVID-19 sequelae are also a risk factor for both problems. However, sequelae exert their effect on work impairment mainly via psychological distress, implying the importance of psychological monitoring and support after discharge. Future research with experimental design should be conducted to develop effective intervention programs to prevent or lessen psychological distress and work impairment in COVID-19 survivors.

## Supplementary Information

Supplementary Information.

## Data Availability

The datasets used and analyzed during the current study are available from the corresponding author on reasonable request.
