# Supplementary material for: The role of discrimination in the relation between COVID-19 sequelae, psychological distress, and work impairment in COVID-19 survivors
Source: Sci Rep. 2022 Dec 23;12:22218. doi: 10.1038/s41598-022-26332-6 (PMC9782263; doi:10.1038/s41598-022-26332-6)
Supplement: Supplementary file 1 — Supplementary Information. [file 41598_2022_26332_MOESM1_ESM.pdf]

Supplementary Table 1. Details of sequelae reported by participants

Sequela	n (%)
Cough	66 (21.4)
Fatigue	62 (20.1)
Olfactory dysfunction	59 (19.1)
Dysgeusia	50 (16.2)
Dyspnea	44 (14.2)
Headache	26 (8.4)
Excess sputum	23 (7.4)
Alopecia	17 (5.5)
Palpitations	15 (4.9)
Loss of concentration	13 (4.2)
Dry eye or mouth	12 (3.9)
Nasal discharge	12 (3.9)
Dizziness	12 (3.9)
Myalgia	11 (3.6)
Anorexia	11 (3.6)
Sore throat	10 (3.2)
Chest pain	10 (3.2)
Arthralgia	7 (2.3)
Insomnia	6 (1.9)
Stomach pain	4 (1.3)
Ocular hyperemia	1 (0.3)

Symptoms are shown in order from highest to lowest frequency.

Supplementary Table 2. Multivariate association between psychological distress and participant characteristics in those who recovered from mild COVID-19

variable		OR (95%CI)	p
Age	Ref: <40		
	40-59	0.79 (0.42-1.46)	0.45
	≥60	1.46 (0.65-3.30)	0.36
Sex	Ref: male	2.36 (1.36-4.08)	0.002
Sequelae		1.80 (1.00-3.25)	0.05
Time period	Ref: prior to 3/1/2021	1.94 (1.08-3.50)	0.006
Exposure to discrimination		2.48 (1.29-4.77)	0.02
No support		0.65 (0.27-1.54)	0.33
Follow-up time		1.00 (0.99-1.01)	0.66

OR: odds ratio, CI: confidence interval

Supplementary Table 3. Multivariate association between work impairment and participant characteristics in those who recovered from mild COVID-19

Variable		OR (95%CI)	p
Age	Ref: <40		
	40-59	1.22 (0.65-2.29)	0.53
	≥60	1.54 (0.67-3.56)	0.31
Sex	Ref: male	1.47 (0.84-2.55)	0.18
Sequelae		2.04 (1.12-3.71)	0.02
Time period	Ref: prior to 3/1/2021	2.96 (1.51-5.80)	0.002
Exposure to discrimination		3.45 (1.89-6.30)	<0.001
No support		0.42 (0.17-1.06)	0.07
Follow-up time		1.00 (0.997-1.012)	0.26

OR: odds ratio, CI: confidence interval

Supplementary Fig. 1 Mediation analysis on the relationship between COVID-19 sequelae and work impairment through psychological distress in participants who recovered from mild COVID-19

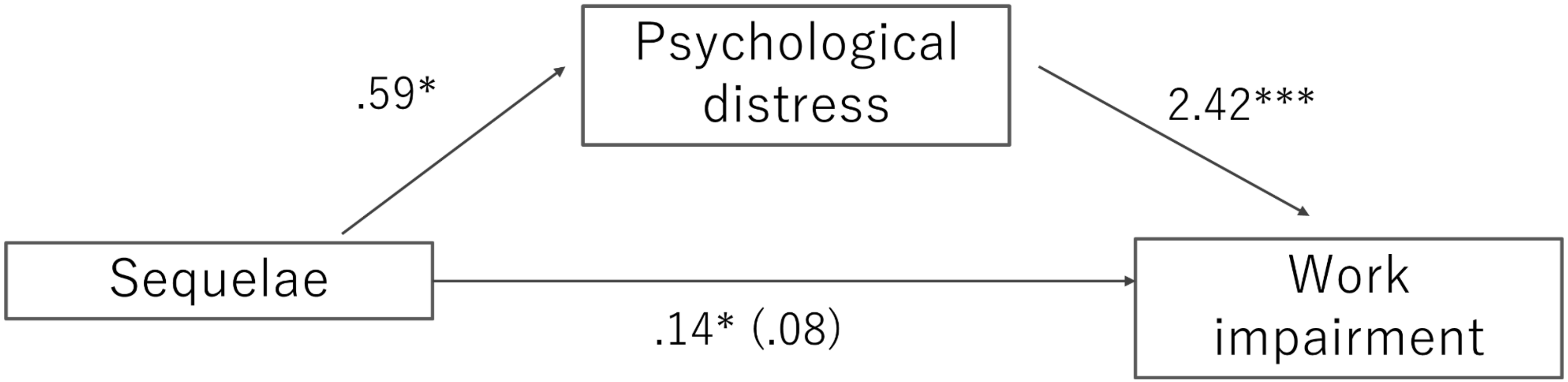

The number between sequelae and psychological distress and the number between psychological distress and work impairment are the effects (unstandardized coefficients) between these elements assuming a causal relationship shown as an arrow.

The numbers between sequelae and work impairment are the total and the direct effects between sequelae and work impairment (the direct effect is in the parenthesis).

* indicates p values < 0.05, ** <0.01, *** <0.001.
